# Key data for outbreak evaluation: building on the Ebola experience

**DOI:** 10.1098/rstb.2016.0371

**Published:** 2017-04-10

**Authors:** Anne Cori, Christl A. Donnelly, Ilaria Dorigatti, Neil M. Ferguson, Christophe Fraser, Tini Garske, Thibaut Jombart, Gemma Nedjati-Gilani, Pierre Nouvellet, Steven Riley, Maria D. Van Kerkhove, Harriet L. Mills, Isobel M. Blake

**Affiliations:** 1 Medical Research Council Centre for Outbreak Analysis and Modelling, Department of Infectious Disease Epidemiology, School of Public Health, Imperial College London, London W2 1PG, UK; 2 Oxford Big Data Institute, Li Ka Shing Centre for Health Information and Discovery, Nuffield Department of Medicine, University of Oxford, Oxford OX3 7FZ, UK; 3 Centre for Global Health, Institut Pasteur, 25-28 Rue du Dr Roux, 75015 Paris, France; 4 MRC Integrative Epidemiology Unit, School of Social and Community Medicine, University of Bristol, Bristol BS8 2BN, UK; 5 School of Veterinary Sciences, University of Bristol, Bristol BS40 5DU, UK

**Keywords:** West African Ebola epidemic, epidemic, mathematical modelling, outbreak response, data, public health

## Abstract

Following the detection of an infectious disease outbreak, rapid epidemiological assessment is critical for guiding an effective public health response. To understand the transmission dynamics and potential impact of an outbreak, several types of data are necessary. Here we build on experience gained in the West African Ebola epidemic and prior emerging infectious disease outbreaks to set out a checklist of data needed to: (1) quantify severity and transmissibility; (2) characterize heterogeneities in transmission and their determinants; and (3) assess the effectiveness of different interventions. We differentiate data needs into individual-level data (e.g. a detailed list of reported cases), exposure data (e.g. identifying where/how cases may have been infected) and population-level data (e.g. size/demographics of the population(s) affected and when/where interventions were implemented). A remarkable amount of individual-level and exposure data was collected during the West African Ebola epidemic, which allowed the assessment of (1) and (2). However, gaps in population-level data (particularly around which interventions were applied when and where) posed challenges to the assessment of (3). Here we highlight recurrent data issues, give practical suggestions for addressing these issues and discuss priorities for improvements in data collection in future outbreaks.

This article is part of the themed issue ‘The 2013–2016 West African Ebola epidemic: data, decision-making and disease control’.

## 1. Introduction

Detection of a new infectious disease outbreak requires rapid assessment of both the clinical severity and the pattern of transmission to plan appropriate response activities. Following the subsequent roll-out of interventions, continued evaluation is necessary to detect reductions in transmission and assess the relative impact of different interventions. Surveillance data are crucial for informing these analyses, and directly determine the extent to which they can be performed.

Despite the unprecedented scale of the 2013–2016 West African Ebola epidemic [1,2], detailed data were collected during the outbreak, which proved invaluable in guiding the response [3–10]. Multiple studies have already considered the lessons to be learned from the Ebola experience with respect to coordinating international responses to health crises, strengthening local health systems and improving clinical care and surveillance tools [11–23]. Here we discuss what can be learned to improve real-time epidemiological assessment in future outbreaks via improved data collection and analyses, building on similar contributions after other epidemics [24–26]. We focus on efforts to reduce and interrupt transmission. First, we outline analyses that are essential to inform response activities during different stages of an epidemic. Second, we detail the various types of data needed to perform these analyses, with examples from the Ebola experience. Third, we summarize the successes and challenges of data collection experienced during this outbreak, and the implications this had for answering key public health questions. Fourth, we suggest improvements that could be implemented in future outbreaks, again drawing from the Ebola experience. Finally, we discuss issues related to availability of data and analyses (box 1).

Box 1. **Recommendations for collecting and using data for outbreak response.**

1. *Collecting relevant data*

Data need to be collected at each of the three levels:

— *individual level*: detailed information about cases
— *exposure level*: information about exposure events that may have led to transmission
— *population level*: characteristics of the population(s) in which the outbreak is spreading and the interventions carried out in the population(s)

Although some data will be context-specific, others, in particular at the population level, will be useful for a wide range of epidemics, and should be routinely and centrally collected in preparation for the next outbreak.

2. *Optimizing data quality*

Having a general framework ready in advance of the next outbreak will facilitate:

— *quality and timeliness* of data
— *centralization and harmonization* of data at all three levels
— *preparedness to deploy* training material, personnel and logistical resources

There is substantial room for improvement in the quality of data collected in an epidemic context, particularly for emerging pathogens.

3. *Ensuring adequate data availability*

Data need to be shared ensuring a balance between the following considerations:

— *ethical*: protecting anonymity while ensuring data are sufficiently detailed to be useful
— *scientific*: wide data access is desirable to promote independent analyses; however, mechanisms must be in place for systematic comparison of results
— *practical*: deciding on a data format for sharing, on who will be responsible for data cleaning and on how various roles will be acknowledged

Discussions and decisions relating to data sharing remain ongoing and guidelines should be agreed on in advance of the next outbreak.

4. *Analysing data and reporting results in an appropriate manner*

The scientific community should agree on guidelines for epidemic modelling and analyses (such as those in place for reporting experimental studies), such as:

— *assumptions* underlying analyses should be clearly stated
— *sensitivity* to these assumptions should be tested
— *uncertainty* in results should be adequately explored and reported

These are particularly relevant for reporting epidemic projections.

## 2. Key public health questions and corresponding analyses

**Figure 1.**
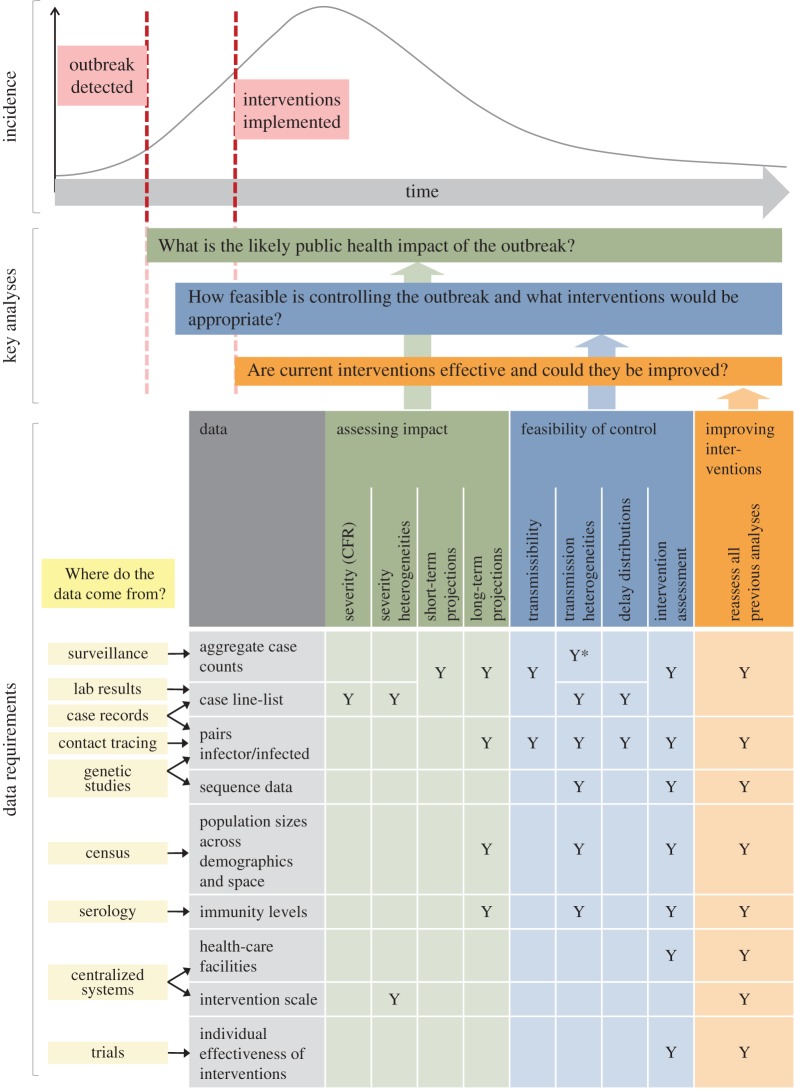
Schematic illustrating the data needed to answer questions at different stages of the epidemic to inform the response. Asterisk indicates that analysis is only possible if aggregate counts are stratified. Footnote: Where two cells in a column are merged, either or both types of data may be used. CFR, case fatality ratio.

Key public health questions for any emerging infectious disease outbreak are the following: (i) What is the likely public health impact of the outbreak? (ii) How feasible is controlling the outbreak and what interventions would be appropriate? (iii) Are current interventions effective and could they be improved? Here, we describe statistical and mathematical analyses that facilitate epidemic response planning, focusing on these questions (figure 1). In this section, we take a general view, as these questions are recurring during most, if not all, outbreaks. We provide examples from the West African Ebola epidemic in subsequent sections.

### (a) What is the likely public health impact of the outbreak?

A key issue in the early phase of an epidemic is to determine the potential impact of the outbreak in terms of clinical severity and the likely total number of cases over different time periods.

#### (i) Severity

The severity of a pathogen is often characterized by the case fatality ratio (CFR), the proportion of cases who die as a result of their infection. Estimating the CFR during an outbreak can be challenging due to inconsistent case definitions, incomplete case reporting and right-censoring of data [27–29]. In particular, it is critical to know the proportion of cases for whom clinical outcome is unknown or has not been recorded, which is typically easier to assess using detailed case data rather than aggregated case counts [27]. The CFR may differ across populations (e.g. age, space, treatment); quantifying these heterogeneities can help target resources appropriately and compare different care regimens. For less severe emerging pathogens, the case definition typically only encompasses a small fraction of all infected individuals, and hence the infection fatality rate (i.e. the proportion of infected individuals who die, rather than the proportion of cases who die—as per the case definition, which may not be equivalent to infection) may be a more useful measure of severity [30].

#### (ii) Short- and long-term incidence projections

Short-term impact of an outbreak can be assessed by predicting the number of cases that will arise in the next few days or weeks. This is particularly relevant for evaluation of immediate health-care capacity needs. Projections of future incidence and estimates of the doubling time (the time taken for the incidence to double) can be obtained by extrapolating the early time series of reported cases either obtained from aggregated surveillance data or calculated from individual records [31]. These projections typically rely on the assumption that incidence initially grows exponentially [31]. They are subject to uncertainty, which increases the further one looks into the future. Quantifying and appropriately reporting such uncertainty and underlying assumptions are crucial [22,25,32,33]. Overstating uncertainty can lead to inappropriately pessimistic projections, which may in turn be detrimental to the control of the outbreak [34]. On the other hand, understating uncertainty prevents policymakers from making decisions based on the whole spectrum of possible impacts. Some studies have already discussed how to find the balance between these two extremes [35–37]. Here, we propose two simple rules of thumb for projecting case numbers. First, projections should not be made for more than two or three generations of cases into the future. Second, central projections should be shown together with lower and upper bounds. In the future, the modelling community should agree on guidelines for reporting epidemic projections, as are already in place for reporting experimental studies [38].

A number of factors can lead to incidence not growing exponentially. In particular, this happens once herd immunity accumulates, if population behaviour changes or as a result of the implementation of interventions. Dynamic transmission models, which account for saturation effects, can be used to assess the long-term impact of the outbreak such as predicting the timing and magnitude of the epidemic peak or the attack rate (final proportion of population infected) [39,40]. However, these models are hard to parametrize as they require information on population size and immunity, interventions (if any) and potential behavioural changes over time, all of which may be subject to uncertainty [41,42]. We discuss these issues in more detail in §3. Projecting longer term is likely to be associated with a large degree of uncertainty and these projections may be more useful for evaluating qualitative trends and evaluating intervention choices rather than predicting exact case numbers.

### (b) How feasible is controlling the outbreak and what interventions would be appropriate?

Interventions to reduce transmission can include community mobilization, quarantine, isolation, treatment or vaccination. The potential success of these interventions is determined by general characteristics of the disease such as overall transmissibility and how this varies across populations [43]. Furthermore, certain types of interventions may be more or less appropriate depending on the natural history of the disease and the context of the epidemic.

#### (i) Overall transmissibility

The transmissibility of a pathogen determines the intensity of interventions needed to achieve epidemic control [44]. The parameter most often used to quantify transmissibility is the reproduction number (*R*), the mean number of secondary cases infected by a single individual. This parameter has an intuitive interpretation: if *R* > 1, then the epidemic is likely to grow, whereas if *R* < 1, the epidemic will decline [44,45]. The final attack rate (proportion of the population infected) of an epidemic also depends on the value of *R* at the start of an epidemic (termed *R*_0_ if the population has no immunity). *R* can be estimated from the incidence of reported cases, given knowledge of the serial interval distribution (i.e. distribution of time between symptom onset in a case and symptom onset in his/her infector; see §2b(iii)) [46–49].

#### (ii) Transmission heterogeneities

Heterogeneity in the number of secondary cases generated by each infected individual affects epidemic establishment and the ease of control. Greater heterogeneity reduces the chance of an outbreak emerging from a single case [50]. However, this heterogeneity can make an established outbreak hard to control using mass interventions, as a single uncontrolled case can generate a large number of secondary cases [50]. Conversely, heterogeneity in transmission may provide opportunities for targeting interventions if the individuals who contribute more to transmission (because of environmental, behavioural and/or biological factors [51–53]) share socio-demographic or geographical characteristics that can be identified [50,54]. Reconstruction of transmission trees (who infects whom) can provide an understanding of who contributes more to transmission. This can be done using detailed case investigations and/or using genetic data [55–58].

Environmental, behavioural and biological factors may also lead to groups of individuals being disproportionately more likely to acquire infection (e.g. children during influenza outbreaks [53,59] or health-care workers (HCWs) during Ebola outbreaks [60,61]). To identify whether such groups exist and target them appropriately, the proportion infected in each group must be estimated. This requires population size estimates for the different groups, which may be difficult to obtain, as we highlight §3c.

Spatial heterogeneity in transmission is particularly interesting to assess as it can inform the targeting of surveillance and interventions to the geographical areas most at risk. Phylo-geographical studies based on genetic data can improve understanding of the geographical origins of the outbreak, identify and characterize sub-outbreaks and quantify whether transmission is very local or travels large distances [22,62,63]. Results of such analyses can be used to determine the appropriate spatial scale of control measures. Spatially explicit epidemic models can also be used to quantify the risk of exportation of the infection from one place to another. This can help public health officials to tailor prevention and control resources to the level of risk likely to be experienced by a given area. Such models typically require detailed data on mobility patterns and immunity levels of the populations in the areas of interest [64–66].

#### (iii) Delay distributions

Disease natural history fundamentally affects outbreak dynamics. The generation time distribution (i.e. distribution of time between infection of an index case and infection of its secondary cases) determines—with the reproduction number—the growth rate of an epidemic [67]. Most commonly, the generation time distribution is estimated from data on the serial interval distribution of an infection—the delay between symptom onset in a case and symptom onset in his/her infector. Other delays between events in the natural history of infection (e.g. exposure, onset of symptoms, hospitalization and recovery or death) also affect disease transmission or have implications for control [43,67]. Delays from symptom onset to recovery (or death) will determine the required duration of health-care and case isolation. The incubation period (the delay between infection and symptom onset of a case) and the extent to which infectiousness precedes symptom onset will determine the feasibility and effectiveness of contact tracing or prophylaxis [43]. Estimating these delay distributions requires detailed data on individual cases and their exposure, e.g. through transmission pairs identified in household studies [43].

#### (iv) Intervention choice

Other analyses can also help refine the type of interventions that should be considered. Ecological associations between transmissibility (measured by *R*) at a fine spatio-temporal scale and any covariate measured at the same scale, may be of interest. For instance, analyses of the West African Ebola epidemic showed that districts with lower reported funeral attendance and faster hospitalization experienced lower transmissibility, highlighting the effectiveness of promoting safe burials and early hospitalization [10].

However, interpreting the results of such analyses can be challenging, as they might be prone to bias and confounding. Efficacy (which measures the impact of an intervention under ideal and controlled circumstances) and effectiveness (which measures the impact of an intervention under real-world conditions) of an intervention (e.g. vaccine) are best measured in a trial setting (either individual- or cluster-randomized [68–70]). However, performing trials to evaluate the comparative impact of different multi-intervention packages is impractical. Dynamic epidemic models, where the interventions of interest can be explicitly incorporated, allow the impact of such intervention packages to be predicted [71]. However, outputs of such models are strongly determined by the underlying assumptions and parameter values. Hence they require careful parametrization, supported by data such as intervention efficacy and the size, infectivity, susceptibility and mixing of different groups [72,73]. These parameters may not be straightforward to estimate, as we discuss in §3 using examples from the West African Ebola epidemic.

Another factor determining the appropriate choice of interventions is their cost, combinations of interventions with higher effectiveness at lower cost (i.e. higher cost-effectiveness) being preferable. Economic analyses combined with mathematical models can help to evaluate the optimal resource allocation among both current available interventions and potential new interventions, accounting for development and testing costs for the latter. Indirect costs, e.g. those associated with a restricted workforce following school closures [74], or trade limitations from air-travel restrictions [75], also need to be considered. While economic analyses have played an important role in designing optimal intervention packages for endemic diseases such as HIV and malaria [76,77], such analyses are more difficult to perform during an epidemic, when cost data might be unavailable and uncertain, costs may vary rapidly over time and ethical considerations suggest interventions should be implemented immediately.

### (c) Are current interventions effective and could they be improved?

Tracking changes in estimates of key epidemiological parameters over the course of an outbreak enhances situational awareness. It also allows the impact of interventions to be assessed as they are implemented, although disentangling the effects of different interventions carried out simultaneously may be challenging.

## 3. Data requirements

Obtaining reliable estimates of the epidemiological parameters detailed above requires a wide range of data, such as incidence time series and detailed case information (figure 1). Here, we explain how these can be obtained from various sources, with the objective to help improve data collection systems in preparation for future outbreaks. We use Ebola as a specific example throughout this section, commenting on the strengths and limitations of the data collected during the West African epidemic.

We distinguish data needs at the individual level, the exposure level and the population level.

### (a) Individual-level data: case line-list

Simple analyses can be performed solely using incidence time series, from surveillance designed to capture aggregate case counts. However, individual case reports provide much richer information, essential to estimate many of the key parameters outlined above (e.g. characterization of delay distributions). Such data are typically stored in a case database or ‘line-list’—a table with one line containing individual data for each case. The more data recorded on each case, the more detailed the analyses can be. In the Ebola epidemic, demographic characteristics, spatial location, laboratory results and clinical details such as symptoms, hospitalization status, treatment and outcome, and dates associated with these were reported for at least a subset of cases. The appropriate information to collect may vary depending on the disease: for Ebola, dates of isolation and funeral were relevant. Comprehensive demographic information can be used to determine risk factors for transmission or severity of infection and to project case numbers stratified by demographic characteristics. Detailed information also helps to identify and merge any duplicate entries in a line-list, which may occur when the same person visits multiple health centres over the course of illness, for instance. Finally, information on how each case was detected—for example, through hospitalization, or via contact tracing—can aid assessment of how representative the data are and allow subsequent adjustment for bias [28]; however, this was not available for Ebola.

Cases in the line-list should be classified using standardized case definitions, which is sometimes difficult for outbreaks of new pathogens, or where different case definitions are provided by different organizations (e.g. World Health Organization (WHO) and US Centers for Disease Control and Prevention (CDC)) [78]. For the Ebola response, although the WHO released official case definitions of confirmed, probable and suspected Ebola cases [79], different countries adopted different testing strategies, thereby limiting the opportunity for inter-country comparison. For example, in Guinea deceased individuals were not tested for Ebola, meaning these individuals were never classified as confirmed cases, unlike in Liberia and Sierra Leone. Encouraging use of a consistent testing protocol and case definition, and ensuring transparency in what is used where and when, would improve the validity of subsequent analyses*.*

Laboratory testing of clinical specimens is key for confirming cases and test results should be linked to the line-list. Understanding diagnostic test performance in field conditions is important; cross-validation of diagnostic sensitivity and specificity between laboratories is useful to assess the extent to which observed differences in case incidence may be explained by variations in laboratory conditions and practices.

In addition, recording raw test results with the case classification may help evaluation of diagnostic performance. Ebola cases were defined as confirmed cases once Ebola virus RNA was isolated from clinical specimens using reverse transcription polymerase chain reaction (RT-PCR [79]). Within each of the main affected countries, field laboratories were established [80,81] to enable prompt diagnosis of cases. Recent evidence suggests that the large fluctuations in temperature and humidity to which these laboratories were subjected reduced the test performance compared with manufacturer evaluation reports [82].

During the West African Ebola epidemic, the case line-list contained a large quantity of data collected from reported cases. The information allowed estimation of the CFR, the incubation period distribution (and evaluation of differences in these by age and gender) and the reproduction number [3,4,6]. Projections of the likely scale of the outbreak were also made, either from the line-list or from aggregated case counts [3,8,83,84]. There were regular data updates [5], with a total of over 19 000 confirmed and probable cases reported, which allowed analyses to be updated as more data became available.

### (b) Exposure-level data

Data on exposures allow cases to be linked to their potential sources of infection, and hence provide a better understanding of transmission characteristics.

#### (i) Transmission pairs

The relevant modes (e.g. airborne, foodborne) and pathways (e.g. animal–human, human–human) of transmission may be identified using information on exposure reported by cases. Cases can report contact both with sick individuals (their potential source of infection) and healthy individuals they have contacted since becoming ill (who may need to be traced and monitored as potential new cases). These data will be more informative if the majority of infections are symptomatic (and hence easily identifiable), if individuals are mostly infectious after the onset of symptoms [43] and if the time window over which exposures and contacts are monitored is as long as the upper bound of the incubation period distribution. If exposure information is collected with enough demographic information to allow record linkage, these backward and forward contacts can be identified in the case line-list, defining transmission pairs. Depending on the mutation rate of the pathogen, genetic data can also be used to identify or confirm these epidemiological links [56,85]. The increasing availability of full genome pathogen sequences offers exciting prospects in that respect. Some genetic sequence information was available during the Ebola epidemic, but most sequences could not be linked to case records, limiting the use of sequence data in this context. On the other hand, individuals who were named as potential sources of infection could often be identified in the case line-list, although this process was hindered by non-unique names and limited demographic information collected on the potential sources [10]. These exposure data were used to characterize variation in transmission over the course of infection [10], and to estimate the serial interval and the incubation period [3]. The upper bound of the incubation period distribution was estimated to be 21 days [3], which supported monitoring contacts for up to three weeks.

#### (ii) Transmission studies

Studies of transmission in well-defined, small settings such as households are useful to quantify asymptomatic transmission, infectivity over time and the serial interval as they capture explicitly the number and timing of secondary cases. Additionally, these studies can estimate the secondary attack rate (the proportion of contacts of a case who become infected within one incubation period), which can be used to characterize heterogeneities in transmission of different groups [59]. Estimates of the secondary attack rate have been obtained for the West African Ebola epidemic by reconstructing household data based on information reported by cases, in particular, as part of contact-tracing activities [86,87].

### (c) Population-level or metadata

Although they might not immediately appear as useful as individual- or exposure-level data, metadata are crucial for answering many public health questions.

#### (i) Population sizes

Knowing the sizes of affected populations is important for quantifying the attack rate and informing dynamic transmission models. Census data are likely to be the most reliable source, but may be infrequently collected. Methods based on interpreting satellite imagery [88,89] can inform population size and structure, although demographic stratifications are not always available. For the West African Ebola epidemic, the most recently available age- and gender-stratified population census data were from 1996 in Guinea [90], 2004 in Sierra Leone [91] and 2008 in Liberia [92].

A particular population of interest is HCWs who, due to their contact with patients, are often at high risk of infection and may also be high-risk transmitters. Large numbers of HCWs were infected during the West African Ebola epidemic [60,61]. However, the proportion of HCWs affected at different stages of the outbreak and the relative risk of acquisition for the HCWs compared with the general population could not be estimated since the total number of HCWs was not systematically reported and changed during the course of the outbreak with the scale-up of interventions. Note that, depending on the transmission route, the definition of HCWs may need to include anyone working in a health-care setting who could be at risk (e.g. cleaners may be exposed to bodily fluids).

#### (ii) Mobility

Characterizing population movement is crucial to assessing the risk of exportation of the infection from one place to another. Air-travel data are the most reliable, consistently available and commonly used data source to inform models of long-distance spread [93]. Such data were widely used during the West African Ebola epidemic to quantify the risk of international spread of the disease, and to assess the potential impact of airport screening and travel restrictions on the outbreak [9,94–96].

However, air travel does not cover other population movements that may play an important role in disease spread, e.g. travel by road or on foot in Guinea, Liberia and Sierra Leone and across the porous country borders during the West African Ebola epidemic [97]. Usually, little data are available to directly characterize these typically smaller-scale movements. Gravity models, which assume that connectivity between two places depends on their population sizes and the distance between them, can be used to quantify spatial connectivity between different regions [98–101], and have proved useful to predict local epidemic spread, e.g. for Chikungunya [98]. Such models require data on population sizes and geographical distances.

Recently, mobile phone data have been explored as an alternative source of data on mobility patterns, which could be used to predict spatial epidemic spread [102–104]. However, a number of challenges (in particular related to privacy issues) meant that such data were unavailable to understand the regional and local spread of the West African Ebola epidemic [105]. In addition, inter-country movement is not captured from these data. At a national level, the utility of mobile phone data may depend on whether mobile phone users are representative of the population contributing to transmission, the level of mobile phone coverage in the affected population and whether population movement is likely to remain the same during an epidemic compared to the time period of the data.

#### (iii) Seroprevalence

Assessing seroprevalence in a population affected by an outbreak can provide valuable information on the underlying scale of population exposure and insight into how interventions might be targeted. For instance, if there is pre-existing population immunity prior to an outbreak that varies with age (as was the case in the 2009 H1N1 influenza pandemic), vaccination could be targeted at those with lower pre-existing immunity. Dynamic transmission models incorporating such differences in susceptibility can be used to explore different targeting strategies [106].

Ideally, serological surveys would be undertaken to quantify seroprevalence [107]; however, they are expensive and not performed on a regular basis. In the absence of such data, information on historical outbreaks and vaccine use can sometimes be used to infer seroprevalence [108]. Serological studies performed before and after an epidemic can also be useful to measure the attack rate and the scale of the outbreak, and hence provide information on the level of underreporting during the outbreak [109].

It was widely assumed that the population in West Africa was entirely susceptible to Ebola at the start of this outbreak, with no known previous outbreaks in the area. However, some studies have suggested that there might have been low levels of prior immunity [110].

#### (iv) Recording intervention efforts across time and space

During an outbreak, multiple interventions are often implemented by different groups and organizations. Evaluating the role of interventions in interrupting transmission is important for revising and improving efforts, but it is challenging without detailed quantitative information of what has been implemented where and when [111,112]. Maintaining a systematic real-time record of the different interventions at a fine spatio-temporal scale would help, e.g. the number and location of health-care facilities and their personnel, number of beds, vaccine or treatment coverage and details of local community mobilization. Developing centralized platforms to routinely record such data once a large-scale outbreak is underway is probably unfeasible. However, developing such tools in advance of outbreaks (such as those developed for the Global Polio Eradication Initiative [113] and those recently developed to collect health-care facility data [114]) should be a priority since better information to evaluate intervention policies in real time will allow for more optimal resource allocation.

During the West African Ebola epidemic, many data on interventions were recorded at a local level by some of the numerous partners (e.g. non-governmental organizations (NGOs) and other organizations) involved in the response. For example, some data were collected on the number and capacity of hospitals over time [115] and these were used in a study modelling community transmission to assess the impact of increasing hospital bed capacity [116]. However, the decentralization of the response meant that intervention data were not systematically reported or collated and these data were not shared widely with the research community. A failure to report interventions centrally and systematically can make it difficult to disentangle a lack of intervention effect from a lack of intervention implementation. This can particularly be a problem when numerous groups coordinate their own efforts, making it impossible to draw firm conclusions about interventions. In the absence of detailed data on intervention efforts in West Africa, multiple studies have assessed the combined impact of all interventions in place, by comparing transmissibility in the early phase (with no intervention) to that in the later phases [87,117]. However, this approach provides less compelling evidence of a causal effect and does not disentangle the impact of different interventions performed at the same time, and hence is less informative for future response planning.

#### (v) Quantifying intervention efficacy

Vaccine or treatment trials together with case–control and cohort studies can be useful in assessing the impact of an intervention. For example, during the West African Ebola epidemic there was an urgent need to estimate the efficacy of newly developed vaccines. Trials such as the Guinea *Ebola ça suffit* vaccine trial [118] provided key data on the effectiveness of the rVSV-ZEBOV Ebola vaccine [119,120]. These trials occurred at the tail end of the epidemic and results will be useful in future outbreaks. Statistical power from trials will be maximized by implementing such studies as early as possible in future outbreaks. This will be facilitated if research on diagnostics, drugs and vaccines is promoted between, and not only during, outbreaks, e.g. through new initiatives such as the Coalition for Epidemic Preparedness Innovation [121].

#### (vi) Quantifying data completeness and timeliness

All of the data sources mentioned above are inevitably imperfect; what they are trying to measure is different from what they measure in practice. Quantifying the mismatch between the two is vital to appropriately account for these imperfections.

For instance, case line-lists are likely to contain information on only a proportion of all infected individuals: typically those with symptoms, or those who sought care. The level of reporting may also be influenced by the capacity of the local health systems, which can vary over time and space. During the West African Ebola epidemic, less than a third of cases were estimated to be reported [122] and severe cases were probably over-represented compared to mild cases. At the end of 2014, health-care capacity was exceeded in many parts of Guinea, Liberia and Sierra Leone [14], but new health-care facilities were subsequently built; hence the line-list of cases is likely to be more complete towards the end of the outbreak. Underreporting might also have been higher in this compared to previous Ebola outbreaks, during which the health-care systems were less overburdened. Systematic evaluation of the surveillance system [123] over different spatial units and time periods could help inform the level of underreporting. In addition, joint analysis of genetic sequence and surveillance data can provide insight into the degree of underreporting [124]. Quantifying completeness of, and potential biases in the line-list is important, e.g. to adequately quantify the CFR [28]. Although differences in the CFR were observed across different health-care facilities in the West African Ebola epidemic, it was not possible to determine whether these were due to reporting differences or underlying differences between settings [125].

Similarly, exposure-level data can be incomplete, depending on the available capacity (personnel and resources) to perform contact tracing and the willingness of people to share information on their contacts. Complete data can be used to assess the route of transmission—animal to human or human to human—and the number of cases imported from other locations. If data are incomplete, these estimates may be incorrect.

Population-level data may suffer the same issues. For instance, the recording of intervention efforts (e.g. the number of personal protection equipment (PPE) kits distributed) can differ from the reality of the intervention (e.g. the number of people who used PPE in practice). Quantifying this mismatch is crucial to evaluating the impact of various interventions, and requires dedicated studies, with both qualitative and quantitative components, on the acceptability of and the adherence to given interventions. Such studies have been carried out in the past, e.g. to assess the potential impact of face masks on the risk of influenza transmission in households, or of condom use on the risk of HIV transmission [126–128].

It is also important to quantify delays encountered in the reporting of cases [129]. During the West African Ebola epidemic, there were delays in all databases and disparities between different data sources. In particular, comparison between the line-list of cases and aggregated daily case counts reported by the affected countries highlighted reporting delays in the line-list. As a result, at any point in the outbreak, naive time series of case counts derived from the line-list suggested that the epidemic was declining, due to right-censoring. Comparison between the line-list and reported aggregated case counts (which were more up-to-date) and between successive versions of the line-list allowed the reporting delays to be quantified. Analyses such as the projected incidence could then be adjusted accordingly [5].

## 4. Successes and challenges in data collection and analyses during the West African Ebola epidemic

In summary, the enormous quantity of detailed data collected during the West African Ebola epidemic played an invaluable role in guiding response efforts. However, several analyses could not be performed. Early on, severity, transmissibility and delay distributions were quantified and short-term projections were made, based on the case line-list and/or contact tracing data [3,5,7]. Some heterogeneity in severity and transmissibility could be identified (e.g. by age and viraemia [7,10,116,125,130–132]), but other types of heterogeneity could not be assessed. For example, it was not possible to compare the CFR between hospitalized and non-hospitalized cases, because of biases in the way cases were recorded in the line-list [28]. Long-term projections were extremely challenging due to large uncertainty in population sizes, behaviour changes and changing intervention efforts. In addition, the relative risk of Ebola acquisition for HCWs was difficult to estimate due to the absence of reliable spatio-temporal data on the number of HCWs. Finally, systematic evaluation of interventions was not feasible due to multiple control measures being carried out at the same time, with little central recording of details of each intervention.

## 5. Optimizing data quality

Data collection during the West African Ebola epidemic was possible, in part, due to a pre-existing case investigation form and data management system (Epi Info viral haemorrhagic fever (VHF) application [133,134]). For outbreaks of new pathogens, such systems are not usually in place. The list of data needs we have outlined above, based on our experience during the Ebola epidemic, could serve as a basis for data collection in future outbreaks. Here we outline improvements that could be made to streamline data collection, minimize delays between data collection and data dissemination, and improve data quality during future outbreaks. All of these are necessary for timely analysis to inform the response in real time. We consider this particularly for line-list and exposure data.

### (a) Streamlining data collection and digitalization

Data need to be digitized before they can be analysed; streamlining this process reduces the potential for delays and errors. Using electronic data capture on tablets or phones may reduce delays and errors compared with using paper forms that then require manual digitization, with its contingent issues [135–138]. Electronic questionnaires may not be available at the start of the epidemic, but could be quickly adopted using available tools (e.g. EpiCollect [139] or EpiBasket [140]). Interpretation problems (e.g. abbreviations) and spelling errors could also be minimized at the data collection level by using multiple choice questions rather than free text and at the data entry level by using drop-down menus. For example, for spatial information the choices could be the standard administrative levels used in the country—such as district or county. As response efforts evolve over time, such as the building of new treatment centres, the lists would be updated as required. Similarly, using pop-out calendars to select dates would limit typing errors. Additionally, internal consistency checks could flag problems such as the recorded date of death being before that of symptom onset, and the system could force a manual check before the record is saved.

In the West African Ebola epidemic, data were collected using paper forms (electronic supplementary material, figures S1–S3) and manually entered into an electronic system at local operation centres. Data entry problems were particularly noticeable in clinical dates and free text variables, such as district locations and hospital names, and required considerable cleaning (e.g. to correct spelling errors) [3,5,10].

### (b) Ensuring adequate logistical resources and training

With any data system there is potential for delays in data entry and dissemination due to limitations in personnel and hardware, and logistical constraints in data delivery (e.g. reliable electricity, Internet access and transport). Although the latter limitations are arguably outside the scope of outbreak control, delays from data collection to dissemination could be reduced by increasing training in data entry and providing more data entry facilities where possible. Delays in data entry and release during the Ebola epidemic meant that real-time analyses of the line-list data could not be performed on fully up-to-date data.

### (c) Acknowledging the international nature of the threat

In the world today, an epidemic emerging anywhere is a global threat due to high population connectivity [141,142] and, as such, a response will have to operate across multiple languages. Ideally, the same data entry system would be used in every affected country to allow easy collation into a single case line-list. However, a global response requires careful translation of the questions and the system to ensure they make sense and are identical in every language. Additionally, different languages, dialects or alphabets could be challenging due to differing pronunciations, accents, alternate names or spellings, and these should be acknowledged in the form design. The language barrier was not a major problem during the Ebola response: Guinea had a form in French, while in Sierra Leone and Liberia the form was in English, though there were some minor differences in formulations between the two (for example the English version of the form asked ‘In the past one month prior to symptom onset: did the patient have contact with (…) any sick person before becoming ill’ (see §4 in the form in electronic supplementary material, figure S1), while the French form did not specify ‘before becoming ill’).

### (d) Improving harmonization between databases

Analysis of laboratory results and sequence data can be much more powerful if they can be dated and linked to the epidemiological data recorded for each case. In the early stages of the Ebola response, there were reports that laboratory results could not always be linked to case records as labels were incorrectly written or damaged in transit [81]. Later in the epidemic, case report forms came with pre-printed unique ID barcode stickers to label all records and samples for each case (electronic supplementary material, Form S2). This would be useful if implemented early in future outbreaks, particularly if laboratory tests are not performed at the point of care. Rapid diagnostic tests were developed during the West African Ebola epidemic but not used widely [143,144]; similarly, mobile sequence tests were introduced later in the epidemic [145]: both of these would reduce delays and maximize the potential to link patient data.

## 6. Data availability

We have described a set of data needed to perform analyses to inform the public health response during an outbreak. We have proposed strategies to ensure fast and high-quality data collection, to enable robust and timely analyses. However, such analyses can only be performed if the data are accessible to data analysts and modellers.

### (a) Data access

The West African Ebola epidemic has prompted an ongoing public debate about the ethical, practical and scientific implications of wide data access [14,146–148]. Ethical considerations require removal of data that might compromise anonymity, but such detailed information might be required to answer important public health questions. Mechanisms also need to be found to appropriately acknowledge those who collected and digitalized the data. From a scientific perspective, having several groups analysing the data is desirable, as independent analyses leading to similar results will reinforce their utility for policymaking [3,8]. Such parallelized efforts have been formalized through consortiums of highly experienced groups, e.g. for modelling of HIV [149], influenza [150,151] and malaria [152]. However, if results differ, understanding what assumptions drive these differences may confuse and delay the process of decision-making. A consequence of data sharing, therefore, needs to be an increased emphasis on evidence synthesis, such as systematic reviews of the different analyses. To enable this process, groups should explicitly state all assumptions underlying their analyses and which data they are based on. Like the original analyses, reviews need to be timely to be useful. These issues have partly been addressed through new data availability policies [153]. Furthermore, this process would ideally be performed by a group independent of those performing the primary analyses. Identifying an effective system, appropriate personnel and appropriate recognition for this important role needs to be planned in advance of future outbreaks.

### (b) Data curating and data cleaning

Data from the West African Ebola epidemic required significant cleaning before it could be analysed, and this was necessary for every updated dataset, i.e. every few days during the peak. Were outbreak data to be shared more widely, collaborative or centralized cleaning would be optimal, to avoid repetition and ensure consistency across different groups. If a centralized effort was not possible, regular sharing of the cleaning code and cleaned datasets between groups would facilitate comparison of results. Even better, code could be shared on a collaborative platform such as Github (https://github.com/), leading to a common clean dataset being actively maintained by the scientific community. However, for this process to be effective, a set of best practices would need to be established in advance, such as designing a transparent workflow, establishing a fair distribution of tasks and clarifying how credit will be given for this (often lengthy) task. This is important to avoid duplication of effort and allow effective collaboration. As the process of data sharing is debated further, it is critical that the practicalities of data cleaning are discussed in parallel. Based on our experience, a centralized cleaning platform would be the most effective method.

### (c) Disseminating results of data analyses

Finally, analyses will be most useful if they are shared widely across policymakers, local health teams and other research groups. The format for dissemination could be anything from a report to a scientific publication. Reports can be made available faster and regularly updated but do not undergo the peer review process of publications. Peer review can delay publication, though there are now new platforms for fast-tracking this process [154,155]. At the time of writing, as interest in Zika virus is gaining momentum, there seems to be an encouraging trend to the use of pre-print servers [156,157].

## 7. Discussion

By the very nature of emerging infectious diseases, we do not know which pathogen will emerge next, when or where. There have been many suggestions about how to be better prepared [11–21,24,26]; here, we argue that preparedness should include development of a broad-use data collection system that can be easily and quickly adapted to any disease (in agreement with [11]) as well as the regular collection of population health data in centralized systems. Different infectious diseases may require different types of data [140]: a single approach is not applicable to all diseases. In particular, different interventions may need to be recorded for different diseases.

Data collection and management may need to evolve as the outbreak progresses and/or as more is learnt about the pathogen: the Ebola data collection forms changed late in 2014 to streamline collection and entry when the response effort was almost overwhelmed with cases (electronic supplementary material, figures S1 and S2). Similarly, both for Ebola and the recent Zika outbreak, reports of sexual transmission have led to a broadening of the contact tracing and exposure information collected.

The data collected during the Ebola epidemic allowed many analyses to be performed, which informed the response. However, as in many outbreak situations, it has not been possible to systematically quantify the relative contribution of different interventions (such as safe burials, hospitalization, contact tracing and community mobilization) in reducing transmission. This is because data on where and when these interventions took place were not centrally recorded and released in a timely fashion. Efforts have been made to collate some information from Sierra Leone, Liberia and Guinea [115]; however, this commenced late in the epidemic. As this effort relies on different organizations to contribute data, the submissions are in different formats and are unlikely to provide comprehensive information. In the midst of a global public health crisis resources are often deployed favouring implementation rather than documentation of interventions. However, we would argue that securing some resources to monitor interventions—especially during the early stages—is critical to optimally prioritizing future control efforts.

Some of the data we have suggested to be collected during outbreaks may not be obviously useful at the field or case management level, e.g. detailed demographic characteristics of cases and contacts. Collecting such data costs money and time as well as trained personnel. These three ‘resources’ are limited and, during an epidemic, should be prioritized where their need is greatest. However, from a population perspective, collecting these data may help quantify epidemic impact, assist in the design and evaluation of interventions, and help prevent new infections. Further studies might examine how to appropriately balance these two considerations.

We have built on the Ebola experience to draw up a list of the data needed to assist the response throughout an epidemic, which should help to collect relevant data in a standardized effort in future epidemics. To make the most of these data, epidemiologists and modellers should work now to develop tools to automatically clean, analyse and report on the data in a more timely and robust manner [158]. Based on critical review of past outbreak analyses, future studies could flag common methodological mistakes and propose good practice [159]. This includes clearly stating all assumptions underlying a model or analysis and ensuring that parametrization is either directly informed by relevant data or has appropriate sensitivity analyses, with corresponding uncertainty clearly reported [32,160].

Improving our ability to respond effectively to the next outbreak will require collaboration between all parties involved in outbreak response: those in the field, epidemiologists, modellers and policymakers as well as the populations affected. Here we have given the data analyst perspective on what data are required to answer important policy questions. It is equally important that other perspectives should be heard to be better prepared for and improve interactions during crises, thereby minimizing the impact of future outbreaks.

## Supplementary Material

Figure S1

## Supplementary Material

Figure S2

## Supplementary Material

Figure S3
