# Supplementary material for: Key data for outbreak evaluation: building on the Ebola experience
Source: Philos Trans R Soc Lond B Biol Sci. 2017 Apr 10;372(1721):20160371. doi: 10.1098/rstb.2016.0371 (PMC5394647; doi:10.1098/rstb.2016.0371)
Supplement: Figure S2 [file rstb20160371supp2.pdf]

# EBOLA CASE INVESTIGATION FORM – Sierra Leone

Date of Case Report: DD/MM/YYYY  Outbreak Case ID: KAI0507234

☐ Patient is a followed contact: ***Convert to CASE in VHF***

**Complete at end of interview:** ☐ suspect ☐ probable ☐ unk

**Patient's Last Name:** ____________ **First Name:** ____________

**Age:** ______ Unit: ☐ Years ☐ Months  Gender: ☐ Male ☐ Female

Patient Status at Time of This Report: ☐ Alive ☐ Dead  ***If dead,*** Date of Death:

**Permanent Residence:**

Head of Household: ____________ Village/Town: ____________

District: ____________ Chiefdom: ____________ Mobile phone #: ____________

**Patient's Occupation:**

☐ Healthcare worker (includes anyone involved with the patient: nurse, ambulance driver, hospital cleaner, etc.)

Position: ____________ Healthcare facility: ____________.

☐ Other; please specify occupation: ____________.

**Location Where Patient Became Ill:**

Village/Town: ____________ District: ____________ Chiefdom: ____________

**Date Patient First Became Sick:** DD/MM/YYYY

**Read each one aloud and mark an answer for every symptom occurred during this illness (not only right now):**

| | | | | | | | |
|---|---|---|---|---|---|---|---|
| Fever | ☐ Yes | ☐ No | ☐ Unk | Headache | ☐ Yes | ☐ No | ☐ Unk |
| Vomiting/nausea | ☐ Yes | ☐ No | ☐ Unk | Difficulty breathing | ☐ Yes | ☐ No | ☐ Unk |
| Diarrhea | ☐ Yes | ☐ No | ☐ Unk | Difficulty swallowing | ☐ Yes | ☐ No | ☐ Unk |
| Conjunctivitis (red eyes) | ☐ Yes | ☐ No | ☐ Unk | Skin rash | ☐ Yes | ☐ No | ☐ Unk |
| Intense fatigue/weakness | ☐ Yes | ☐ No | ☐ Unk | Hiccups | ☐ Yes | ☐ No | ☐ Unk |
| Anorexia/loss of appetite | ☐ Yes | ☐ No | ☐ Unk | Unexplained bleeding | ☐ Yes | ☐ No | ☐ Unk |
| Abdominal pain | ☐ Yes | ☐ No | ☐ Unk | If yes, please specify: ____________ | | | |
| Muscle pain | ☐ Yes | ☐ No | ☐ Unk | Other symptoms: | ☐ Yes | ☐ No | ☐ Unk |
| Joint pain | ☐ Yes | ☐ No | ☐ Unk | If yes, please specify: ____________ | | | |

**At the time of this report, is the patient hospitalized or being admitted to the hospital?** ☐ Yes ☐ No ☐ Unk

If yes, Date of Hospital Admission DD/MM/YYYY

Hospital Name: ____________ District: ____________

Is the patient now, or will he/she soon be, in an Ebola treatment unit? ☐ Yes ☐ No ☐ Unk

If yes, date of admission (or future admission to the ETU (isolation): DD/MM/YYYY

Was the patient hospitalized or visited a clinic previously for this illness (this includes any type of care: pharmacist, traditional healer, etc.)? ☐ Yes ☐ No ☐ Unk

If yes, Dates of Hospitalization: DD/MM/YYYY

Hospital Name: ____________ District: ____________

Uniprint-F_SA

KAI0507234  KAI0507234  KAI0507234

KAI0507234  KAI0507234  KAI0507234

IN THE PAST ONE (1) MONTH PRIOR TO SYMPTOM ONSET:

Outbreak Case ID: KAI0507234

1. Did the patient have contact with a suspected or confirmed Ebola case in the one month before becoming ill?

☐ Yes ☐ No ☐ Unk

*If yes, please complete one line of information for each sick source case:*

| Name of Source Case | Relation to Patient | Date of Last Contact (DD, MM, YYYY) | Village/Town | District | Was the person dead or alive? |
|---|---|---|---|---|---|
| | | / / | | | ☐ Alive<br>☐ Dead<br>Date of Death:<br>/ /<br>(DD,MM,YYYY) |
| | | / / | | | ☐ Alive<br>☐ Dead<br>Date of Death:<br>/ /<br>(DD,MM,YYYY) |

2. Did the patient attend a funeral in the one month before becoming ill? ☐ Yes ☐ No ☐ Unk

| If yes, Name of deceased person | Relation to Patient | Date of Funeral (DD, MM, YYYY) | Village/Town | District | Did the patient participate? (carry or touch the body)? |
|---|---|---|---|---|---|
| | | / / | | | ☐ Yes<br>☐ No |

3. Did the patient travel outside their hometown or village/town before becoming ill? ☐ Yes ☐ No ☐ Unk

If yes, Village: ________ Chiefdom: ________

District: ________ Date(s): __/__/____ - __/__/____

**Case Report Form Completed by:**

Name: ________ Phone: ________ E-mail: ________

Position: ________ District: ________ Health Facility: ________

Information provided by:

☐ Patient ☐ Proxy *If proxy,* Name: ________ Relation to patient: ________

**Patient Outcome Information:**

***Please fill out this section at the time of patient recovery and discharge from the hospital OR patient death.***

**Date Outcome Information Completed:** __/__/____

**Final Status of the Patient:** ☐ Alive/Recovered ☐ Dead

***If the patient has recovered and been discharged from the hospital:***

Hospital discharged from: ________ District: ________

Date of discharge from the hospital: __/__/____

***If the patient is dead:***

Date of Death: __/__/____

Place of Death: ☐ Community ☐ Hospital District: ________

Date of Funeral/Burial: __/__/____

Funeral conducted by: ☐ Family/community ☐ Outbreak burial team

Place of Funeral/Burial: Village: ________ Chiefdom: ________ District: ________

Uniprint-F_SA

**EBOLA Clinical Specimens and Laboratory Testing**

Outbreak Case ID: KAI0507234

## Sample 2

**Date of Specimen Collection:** DD/MM/YYYY

**Patient's Last Name:** ______________ **First Name:** ______________

**Age:** _______ Unit: ☐ Years ☐ Months Gender: ☐ Male ☐ Female

**Permanent Residence:**

Head of Household: ______________ Village/Town: ______________

District: ______________ Chiefdom: ______________ Mobile phone #: ______________

**Date Patient First Became Sick:** DD/MM/YYYY

Specimen/shipping instructions:
- Label sample with **patient name, date of collection,** and **case ID**
- Send sample cold with a **cold/ice pack**, and **packaged appropriately**
- Collect whole blood in a purple top (EDTA) tube green or red top tubes acceptable if purple not available
- **Preferred** sample volume = **4ml** (minimum sample volume = 2ml)

Uniprint-F_SA

Has this patient had a sample submitted previously? ☐ Yes ☐ No ☐ Unk

Sample Type: ☐ Whole blood ☐ Skin biopsy

☐ Post-mortem heart blood ☐ Other specimen type, specify: ______________

TEAR HERE TEAR HERE TEAR HERE TEAR HERE TEAR HERE TEAR HERE TEAR HERE

**EBOLA Clinical Specimens and Laboratory Testing**

Outbreak Case ID: KAI0507234

## Sample 1

**Date of Specimen Collection:** DD/MM/YYYY

**Patient's Last Name:** ______________ **First Name:** ______________

**Age:** _______ Unit: ☐ Years ☐ Months Gender: ☐ Male ☐ Female

**Permanent Residence:**

Head of Household: ______________ Village/Town: ______________

District: ______________ Chiefdom: ______________ Mobile phone #: ______________

**Date Patient First Became Sick:** DD/MM/YYYY

Specimen/shipping instructions:
- Label sample with **patient name, date of collection,** and **case ID**
- Send sample cold with a **cold/ice pack**, and **packaged appropriately**
- Collect whole blood in a purple top (EDTA) tube green or red top tubes acceptable if purple not available
- **Preferred** sample volume = **4ml** (minimum sample volume = 2ml)

Uniprint-F_SA

Has this patient had a sample submitted previously? ☐ Yes ☐ No ☐ Unk

Sample Type: ☐ Whole blood ☐ Skin biopsy

☐ Post-mortem heart blood ☐ Other specimen type, specify: ______________
