# Supplementary figures and images for: Key data for outbreak evaluation: building on the Ebola experience
Source: Philos Trans R Soc Lond B Biol Sci. 2017 Apr 10;372(1721):20160371. doi: 10.1098/rstb.2016.0371 (PMC5394647; doi:10.1098/rstb.2016.0371)

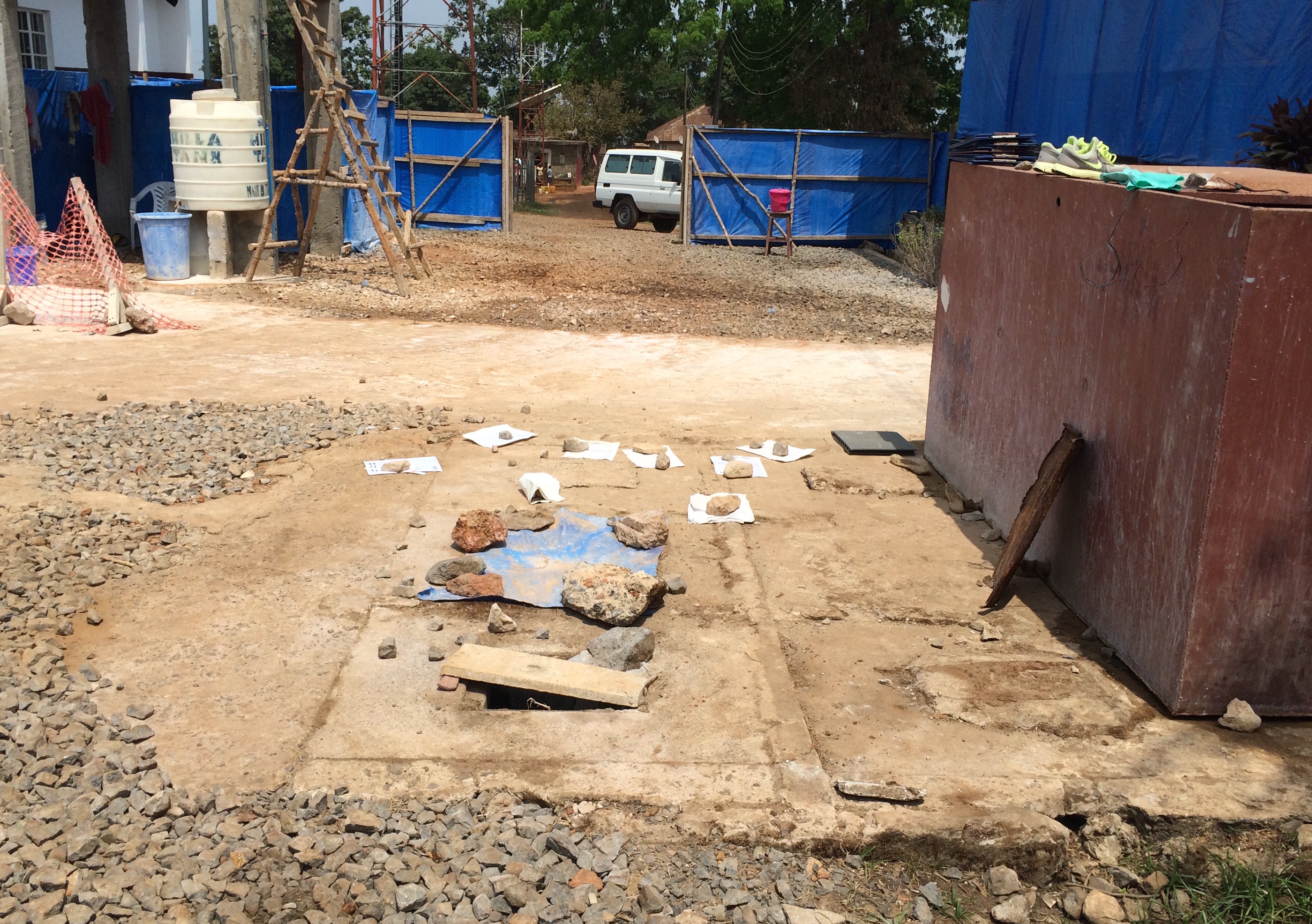

Supplement: Figure S3 [file rstb20160371supp3.jpg]
